# Spatiotemporal tissue temperature during cryoablation using different balloons

**DOI:** 10.1016/j.hroo.2025.06.005

**Published:** 2025-06-16

**Authors:** Akio Chikata, Takeshi Kato, Hiroaki Ide, Tatsuya Fukutani, Shuhei Fujita, Kazuo Usuda, Michiro Maruyama, Kan-ichi Otowa, Takashi Kusayama, Kenshi Hayashi, Masayuki Takamura

**Affiliations:** 1 Department of Cardiology, Toyama Prefectural Central Hospital, Toyama, Japan; 2 Department of Cardiovascular Medicine, Kanazawa University Graduate School of Medical Science, Kanazawa, Japan; 3 Murata Manufacturing Co., Ltd, Kyoto, Japan; 4 Department of Pediatrics, Toyama Prefectural Central Hospital, Toyama, Japan

**Keywords:** Spatiotemporal, Tissue temperature, Cryoablation, AFA-Pro, POLARx FIT

## Abstract

**Background:**

Differences in tissue temperature trends and distribution between two commercially available balloons during cryoablation remain unclear.

**Objective:**

We sought to evaluate spatiotemporal tissue temperatures during cryoablation using different cryoballoons.

**Methods:**

An in vitro pulmonary vein model was constructed from porcine myocardial tissue to simulate pulmonary vein anatomy and venous flow. A multitemperature sensor sheet was placed behind the muscles. After confirming pulmonary vein occlusion, cryoablation was performed using the Arctic Front Advance Pro (AFA-Pro) and POLARx FIT (28 and 31 mm) to evaluate the time course of spatial tissue temperatures.

**Results:**

POLARx FIT 28 mm showed the lowest force for pulmonary vein (5.5 ± 1.0 N for AFA-Pro, 4.0 ± 0.8 N for POLARx FIT 28 mm, and 4.1 ± 0.7 N for POLARx FIT 31 mm, respectively (*P* < .05; POLARx FIT 28 mm vs AFA-Pro), and the lowest tissue temperature after 180 seconds of cryoablation (−44.7 ± 2.3°C for AFA-Pro, −50.1 ± 5.7°C for POLARx FIT 28 mm, and, −44.7 ± 2.4°C for POLARx FIT 31 mm, respectively (*P* < .05; POLARx FIT, 28 mm vs AFA-Pro and POLARx FIT, 31 mm). Multipoint sensor analysis revealed that only POLARx FIT 28 mm achieved a tissue temperature < −50°C, while the POLARx FIT 31 mm created a significantly larger area with a tissue temperature of −30°C or lower.

**Conclusion:**

Among commercially available balloons, the POLARx FIT of 28 mm achieved the greatest tissue temperature drop, whereas the POLARx FIT of 31 mm created the broadest cooled tissue area.

**Key Findings**

▪ Spatiotemporal temperature evaluation in an in vitro model suggested differences in cooling profiles between the two commercially available balloons.
▪ The minimum force required for pulmonary vein occlusion was significantly lower with POLARx FIT 28 mm compared with AFA-Pro.
▪ .POLARx FIT 28 mm achieved the lowest tissue nadir temperature (−50.1 ± 5.7°C), including instances below −50 °C, suggesting the potential for creating deep lesions.
▪ POLARx FIT 31 mm generated the broadest area of cooling below −30°C, indicating a capacity for creating wider lesion sets.

## Introduction

Pulmonary vein (PV) isolation (PVI) is the cornerstone of atrial fibrillation (AF) therapy and is recommended for patients with left ventricular dysfunction or those who are receiving ineffective antiarrhythmic therapy.^1^, ^2^, ^3^ Cryoballoon-based PVI is non-inferior to radiofrequency-based PVI,^4^ and more recently, cryoballoon-based PVI is useful as a first-line treatment for preventing AF recurrence and progression.^5^, ^6^, ^7^ Two different cryoablation systems are currently available. The safety and efficacy of the Arctic Front Advance Cryoballoon (Arctic Front Advance and Arctic Front Advance Pro [AFA-Pro], Medtronic, Inc., Minneapolis, MN) have been thoroughly evaluated, showing excellent acute success rates, lower complication rates, and promising long-term follow-up results.^5^, ^6^, ^7^, ^8^ The newly introduced POLARx Cryoballoon system (Boston Scientific, St Paul, MN) has also been reported to be as effective as the AFA-Pro.^9^, ^10^, ^11^, ^12^, ^13^, ^14^ However, it has been noted that POLARx Cryoballoon may cause greater myocardial and collateral damage compared to AFA-Pro.^12^, ^13^, ^14^

The balloon of the POLARx Cryoballoon system is compliant and capable of maintaining a constant balloon pressure throughout the inflation and freezing cycles, which may improve the occlusion state in various PV anatomies and prevent balloon dislodgement from the PV ostium. The POLARx FIT (Boston Scientific, Marlborough, MA) is a novel size-adjustable cryoballoon system. POLARx was available only in a 28 mm diameter. In contrast, the POLARx FIT can be inflated to 2 diameters (28 mm and 31 mm) using the same procedure.^15^, ^16^, ^17^ This provides larger antral isolation areas and may allow for the treatment of larger pulmonary veins.

Although cryoballoons are effective devices, they are known to have different balloon profiles and cooling temperatures.^9^^,^^10^ Hayashi and collegaues^18^ demonstrated in their in vitro experiments that both balloon temperature and tissue temperature were significantly lower in POLARx compared to the balloon and tissue temperatures in AFA-Pro. However, this was a single-point evaluation rather than a multipoint assessment. The results did not consider the size-adjustable POLARx FIT, and the effect of different balloon sizes on the temperature of biological tissue remains unexplored. Therefore, this study aimed to evaluate the temperature changes in biological tissue at multiple points during cryoablation for each balloon size using an in vitro model that simulates the anatomy and flow of human pulmonary veins.

## Methods

### In vitro setup and construction of a pulmonary vein phantom

**Figure 1:**
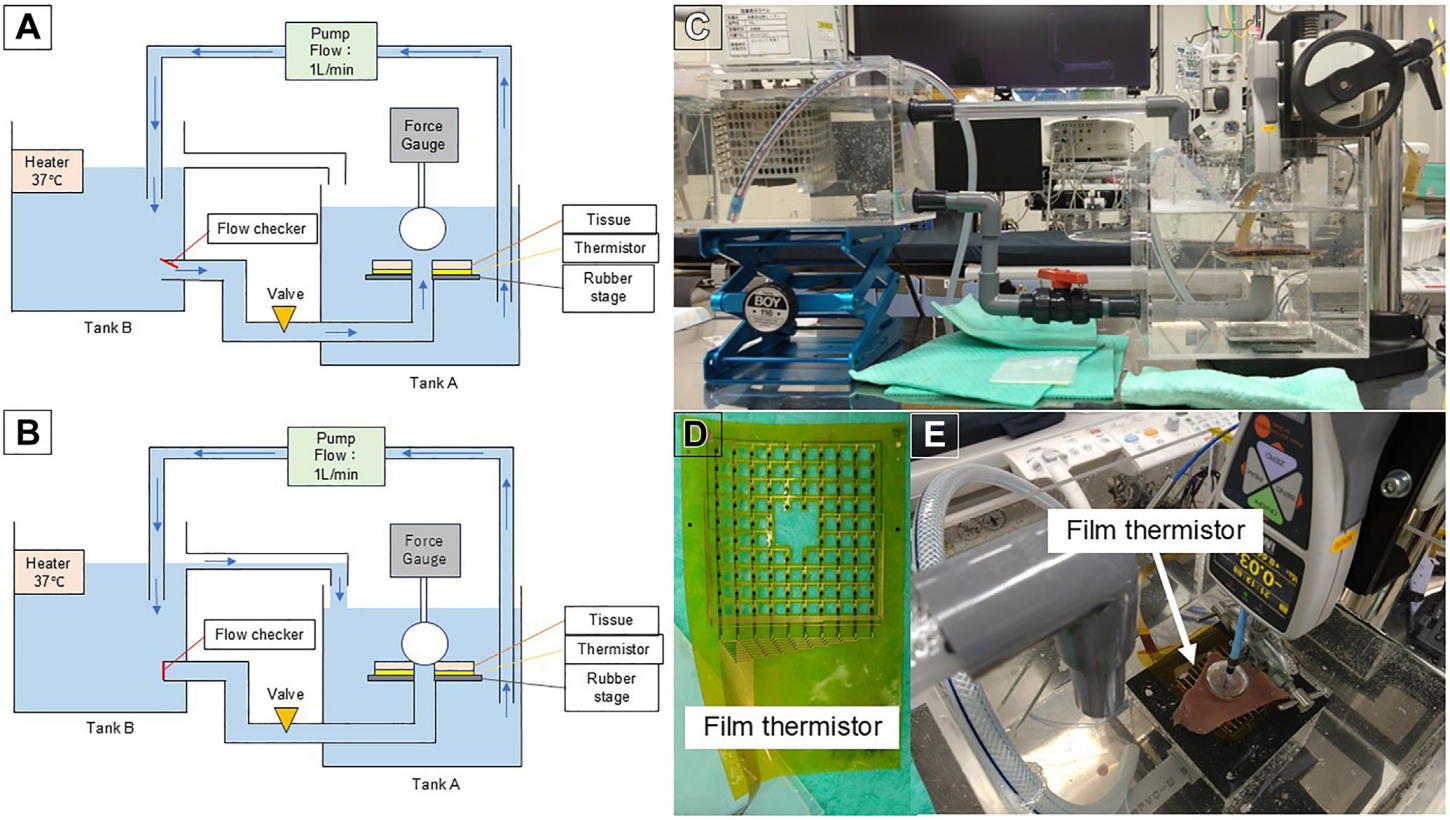
In vitro experimental setup. The simulated pulmonary venous flow was controlled using a pump and valve at 1 L/min. Occlusion was evaluated using the slope change in the Flow Checker. The flow checker had a plastic film fixed at a single point on the upper part of the piping and arranged to block the flow path from Tank B to Tank A. When water flow occurs, the lower end tilts in the direction of the flow; when occluded, it faces directly downward owing to gravity, confirming occlusion (A, B, and C). A multiple-temperature sensor sheet (film thermistors; Murata Manufacturing Co., Ltd. Kyoto, Japan) was attached to myocardial tissue (D and E).

A plastic tank containing 0.9% normal saline, maintained at 37 °C by a circulating water pump, was used for in vitro simulations. A pulmonary vein model was constructed from porcine myocardial tissue and subsequently positioned on a table to simulate pulmonary vein anatomy and venous flow. Based on previous reports, we used a table with a 15 mm diameter hole in the center, reflecting the typical pulmonary vein diameter (Figure 1A, 1B, and 1C). The hole was connected to a 15 mm diameter plastic tube to maintain the flow from the tank. The flow rate was adjusted using a valve to simulate a pulmonary vein flow rate of 1 L/min.^18^, ^19^, ^20^ Fresh porcine hearts were obtained, and ventricular myocardial tissue slices measuring 2–2.5 mm were placed on the designed table (Figure 1E). The thickness of each myocardial tissue slice was measured. Tissue slices were controlled and assigned to each experimental group so that each experiment used tissue slices of comparable thickness. The porcine myocardial tissue used in this study was obtained from commercially available sources. Although no live animals were used, all procedures complied with institutional ethical standards, and the study adhered to the principles of the ARRIVE guidelines and the Guide for the Care and Use of Laboratory Animals

### Evaluation of the force needs for pulmonary vein occlusion by each balloon

The proximal portion of the cryoballoon catheter was attached to a pillar and connected to a force gauge to measure the force exerted by the catheter tip on the tissue (ZTS-50N, IMADA CO., LTD. Aichi, Japan). The force gauge was fixed to an elevating stand, which could be raised and lowered by handling (Figure 1E). Occlusion of the pulmonary vein model by the cryoballoon was confirmed by tilting the flow checker, and the minimum force required for occlusion was recorded simultaneously (Figure 1B).

### Investigation of the relationship between balloon temperature and tissue temperatures at multiple points

A multiple-temperature sensor sheet (film thermistor; Murata Manufacturing Co., Ltd. Kyoto, Japan), adjusted for a measurement range of −70 °C to 0 °C , was attached under the myocardial tissue to measure temperature during cryoablation (Figure 1D and 1E). The cryoballoon and tissue temperatures were recorded during cryoablation. Tissue temperature was evaluated both over time and as a two-dimensional distribution. Eight samples from each porcine myocardial tissue were used. Based on a previous clinical report, cryoablation was performed for 180 seconds, and the freezing cycle was completed.

### Statistical analyses

Statistical analyses were performed using GraphPad Prism software (GraphPad Software, La Jolla, CA). Data are presented as mean ± standard deviation. Data were compared using the Kruskal–Wallis test, Dunn's test, and Bonferroni correction to examine the differences between the 3 groups. All tests were two-sided, and a *P* value < .05 was considered statistically significant.

## Results

### The force needed to occlude the pulmonary vein

The cryoballoon was inflated in a standard manner in a water bath after confirming that the cryoballoon occluded the myocardial tissue using a flow checker (Figure 1B). The minimum force was recorded at this point (Figure 1E). The minimum force for occlusion of the pulmonary vein model with each cryoballoon was 5.5 ± 1.0 N for AFA-Pro, 4.0 ± 0.8 N for POLARx FIT 28 mm, and 4.1 ± 0.7 N for POLARx FIT 31 mm. The POLARx FIT 28 mm had the lowest value. (*P* < .05 vs AFA-Pro). In the case of cryoablation with AFA-pro, the occlusion could be maintained at the same pressure after the start of cryoablation (Table 1).

**Table 1:** Balloon characteristics and tissue temperatures of POLARx FIT and Arctic Front Advance Pro (AFA-Pro)

	AFA-Pro	POLARx FIT	
	n = 8	n = 8	n = 8
Balloon size (mm)	28	28	31
N_2_O fluid flow during freeze (sccm)	7200	7800	8700
Pressure during freesing (psi)	18−20 psi	2.5 psi	7.5 psi
Tissue Thickness (mm)	2.3 ± 0.3	2.3 ± 0.3	2.3 ± 0.2
Mimmum force (N)	5.5 ± 1.0	4.0 ± 0.8	4.1 ± 0.7
Balloon temperature			
Mean Nadir temperature (°C)	−52 ± 1.3	−63.3 ± 2.2∗	−64 ± 1.5∗
Time to −30°C (s)	24.4 ± 1.5	24.9 ± 1.5	23.6 ± 1.1
Time to 0°C (s)	12.3 ± 0.7	25.3 ± 2.5∗	25.4 ± 1.7∗
Tissue temperature distribution			
30 s in cryoablation			
minimum temperature (°C)	−30.0 ± 4.5	−34.2 ± 9.9	−38.8 ± 5.5∗
60 s in cryoablation			
minimum temperature (°C)	−39.5 ± 3.3	−48.6 ± 6.3∗	−43.5 ± 2.1
180 s in cryoablation			
minimum temperature (°C)	−44.7 ± 2.3	−50.1 ± 5.7†‡	−44.7 ± 2.4
10 s after cryoablation			
minimum temperature (°C)	−23.6 ± 1.2	−41.4 ± 5.0∗	−38.6 ± 12.8∗

AFA-Pro = Arctic Front Advance Pro.

∗ *P* < .01 vs AFA-Pro.

† *P* < .05 vs AFA-Pro.

‡ *P* < .05 vs POLARxFIT 31 mm.

### Distribution of balloon and tissue temperature changes for each cryoballoon

The thickness of each tissue slice was not different between the groups (Table 1).

**Figure 2:**
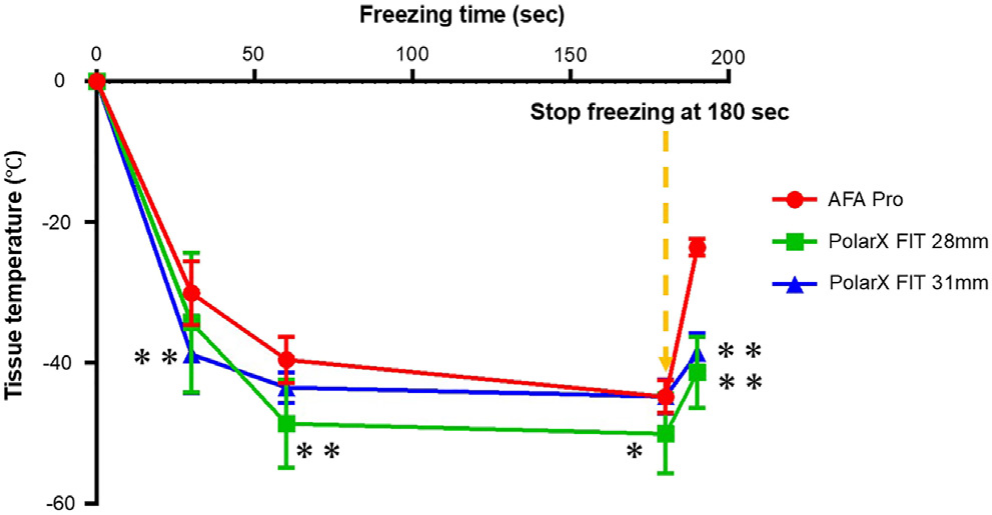
Time course of minimum tissue temperature for each balloon. Cryoapplication was stopped at 180 seconds. *P* < .05 vs AFA-Pro,∗∗ *P* < .01 vs AFA-Pro.

The balloon nadir temperature was significantly lower in POLARx FIT (28 and 31 mm) than in AFA-Pro. A comparison between POLARx FIT balloon sizes showed no significandifference in nadir temperature. Tissue temperature was significantly lower in the POLARx FIT 28 mm group than in the AFA-Pro and POLARx FIT 31 mm groups at 60 and 180 seconds. Thaw time (time to 0°C) was significantly longer in POLARx FIT 28 mm and 31 mm than AFA-Pro, and the tissue temperature was significantly lower in POLARx FIT 28 mm and 31 mm than AFA-Pro at 10 seconds after cryoapplication (Table 1 and Figure 2).

**Figure 3:**
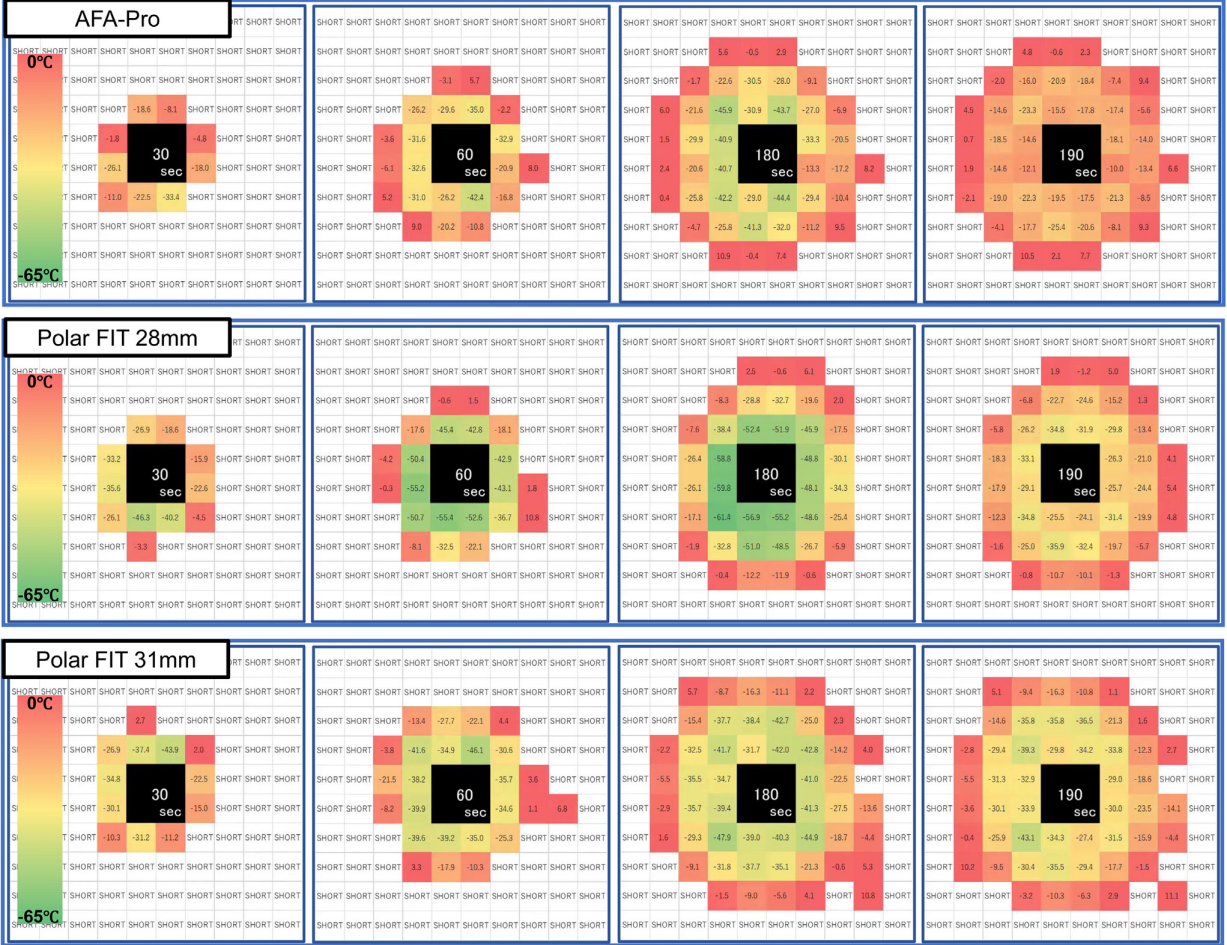
Real time images of tissue temperature at each time point. Temperature distributions at 30, 60, 180, and 190 (10 seconds after freezing stopped) seconds after cryoapplication. The upper panel shows AFA-Pro, the middle panel shows POLARx FIT 28 mm, and the bottom panel shows POLARx FIT 31 mm.

**Figure 4:**
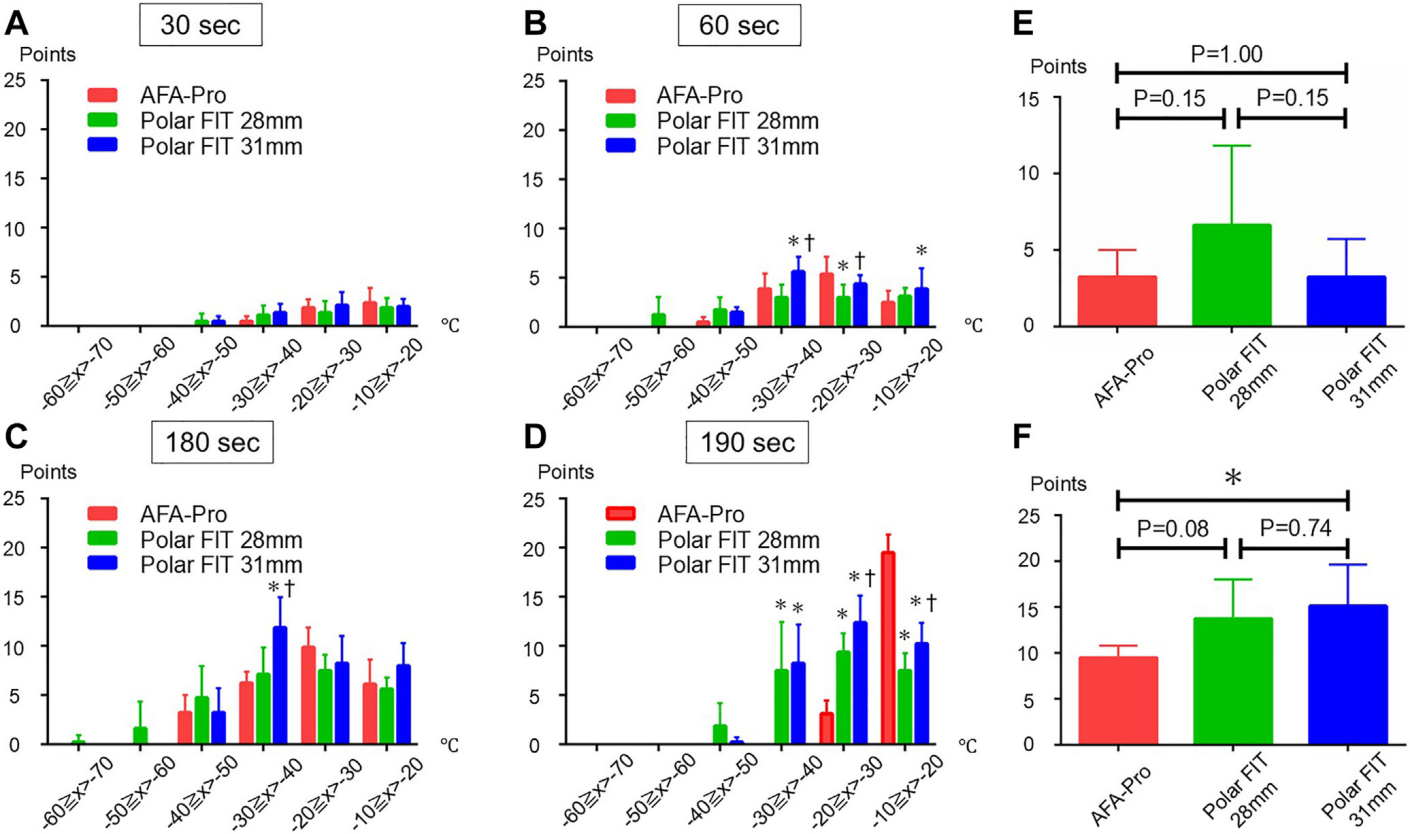
Distribution of tissue temperature at each time point. The distribution of temperatures indicated by each sensor at 30, 60, 180, and 190 (10 seconds after freezing stopped) seconds after cryoapplication (A, B, C, and D). The comparison of the number of sensors shows −40 °C or less at 180 seconds (E) and −30 °C or less at 180 seconds (F). ∗ *P* < .05 vs AFA-Pro, † *P* < .05 vs polar 28 mm.

The POLARx FIT 31 mm balloon showed a decrease in tissue temperature over a wide area, as shown by the tissue temperature distribution over time at multiple points (Figures 3, 4, and Supplemental Videos 1, 2, and 3). Although there was a tendency for more instances of tissue temperature dropping below −40 °C at 180 seconds in the POLARx FIT 28 mm group, the difference was not statistically significant (Figure 4E). Only POLARx FIT 28 mm showed some points where the tissue temperature reached below −50 °C (Figures 3, 4B, 4C, and Supplemental Videos 1, 2, 3). Significantly more points reached −30 °C or lower with the POLARx FIT 31 mm balloon (Figure 4F).

## Discussion

This study evaluated the temperature changes in biological tissue at multiple points during cryoablation with AFA-Pro and POLARx FIT (28 mm and 31 mm) using an in vitro model. Our major findings were as follows: (1) the minimum force for occlusion of the pulmonary vein model was different for each cryoballoon type and size; (2) Compared to the AFA-Pro and POLARx FIT 31 mm, the mean tissue nadir temperature during cryoablation was significantly lower with POLARx FIT 28 mm; and (3) POLARx induced greater and more prolonged tissue temperature reductions over a wider area than AFA-Pro, especially with the POLARx 28 mm, which achieved the greatest tissue temperature drop, and the 31 mm created the broadest cooled tissue area.

The balloon materials and internal pressure may affect the force required to completely occlude the veins. The materials of the 2 cryoballoons were different. POLARx is a semi-elastic thermoplastic material that is more adaptable and compliant than AFA-Pro. Furthermore, the internal pressure of the POLARx Cryoballoon during cryoablation has been reported as 2.5 psi (0.2 atm gauge pressure) for the 28 mm balloon, 7.5 psi (0.6 atm gauge pressure) for the 31 mm balloon, and 18–20 psi (1.2 atm gauge pressure) for AFA-Pro. These differences may indicate that a stronger force is required to achieve occlusion with AFA-Pro than with POLARx.

The N_2_O cooling flow rate for POLARx 28 mm was 7800 standard cubic centimeters per minute (sccm); POLARx 31 mm, 8700 sccm, which was greater than the 7200 sccm flow rate for AFA-Pro. The internal pressure of the POLARx 28 mm was 1.2 atm (absolute pressure), and the POLARx FIT 31 mm was 1.6 atm (absolute pressure), which implies that the boiling point of N_2_O is around −85 °C and −80 °C, respectively, based on the vapor pressure curve. On the other hand, the internal pressure of AFA-Pro was 2.2 atm (absolute pressure), indicating that the boiling point of N_2_O is around −74 °C.^21^ These differences in N_2_O flow rate and internal pressure could affect the tissue nadir temperature and the heterogeneity of the cooling effect.

Rapid cooling, gradual recovery, and low nadir temperatures contribute to cryoenergy-induced tissue injury.^22^^,^^23^ It has been reported that when the tissue temperature drops to about −40 °C, permanent cell damage occurs due to mechanical disruption of protoplasmic structures and cell membranes due to intracellular ice formation.^24^, ^25^, ^26^ In this study, POLARx FIT 28 mm showed the lowest tissue temperatures, while the 28 mm and 31 mm showed gradual recovery. Although the POLARx FIT 28 mm showed a trend toward more instances of tissue temperature dropping below −40 °C at 180 seconds, the difference was not statistically significant. However, only the POLARx FIT 28 mm displayed instances where the tissue temperature dropped below −50 °C. The epicardial tissue temperature during cryoablation is involved in the formation of durable lesions and in the development of collateral tissue damage. Although collateral tissue damage using thermal energy sources has adverse effects, such as esophageal thermal injury and phrenic nerve injury, it also has beneficial effects, such as ganglionated plexus (GP) modification.^27^, ^28^, ^29^, ^30^ When using the POLARx FIT, especially the 28 mm balloon, while the powerful cooling effect allows durable lesions to be created, care needs to be taken to avoid collateral tissue damage. On the other hand, POLARx FIT 31 mm exhibited a significantly larger area below −30 °C, while the area below −40 °C was comparable to AFA-Pro, and no area lowering below −50 °C was observed. Considering the gradual tissue temperature recovery of POLARx FIT, it is estimated that POLARx FIT 31 mm can form lesions comparable to AFA-Pro in wider areas with a comparable risk of collateral damage.

These findings suggest that tailoring balloon selection based on individual patient anatomy and procedural goals may help optimize outcomes. For instance, the POLARx FIT 28 mm may be more suitable in patients requiring high lesion durability and neuromodulation, whereas the POLARx FIT 31 mm and AFA-pro may offer a safer alternative in cases where minimizing the risk of collateral injury is critical, such as when the esophagus or phrenic nerve is in close proximity. Further studies are warranted to assess whether these biophysical differences translate into meaningful differences in long-term efficacy and safety in clinical practice.

### Study limitations

First, the focus was only on temperature distributions, and actual histological changes were not evaluated. In vivo studies with appropriate physiological response will be essential to further validate the histopathological implications of our findings. Second, the pulmonary vein diameter was assumed to be 15 mm; however, different pulmonary vein diameters may affect the balloon contact and tissue temperature. Third, although incomplete pulmonary vein occlusion and mitral regurgitation may affect balloon and tissue temperatures.^20^^,^^31^ Fourth, pericardium is exposed to the thoracic cavity with a low thermal load,^32^ we did not evaluate these factors in this study. Fifth, although our study was conceptually designed in the context of pulmonary vein isolation and atrial fibrillation, the experiments were conducted using ventricular myocardial tissue. While this tissue type differs structurally from atrial myocardium, prior in vitro studies have similarly used ventricular or skeletal muscle models to investigate cryoballoon biophysics.^20^^,^^26^^,^^33^, ^34^, ^35^ These models allow for controlled evaluation of temperature dynamics and lesion formation under defined flow conditions. Nonetheless, extrapolation to atrial tissue must be approached with caution, and our findings should primarily be interpreted as a comparative assessment of cryothermal behavior across balloon systems under standardized in vitro conditions.

### Conclusion

In this experimental model, the spatiotemporal tissue temperatures during cryoablation differed between the two commercially available balloon types and sizes. The POLARx FIT 28 mm exhibited the greatest decrease in tissue temperature. In contrast, the POLARx FIT 31 mm was capable of creating the widest lesions.

## Disclosures

The authors have no conflicts to disclose.

## References

[1] Hindricks G., Potpara T., Dagres N. (2021). 2020 ESC Guidelines for the diagnosis and management of atrial fibrillation developed in collaboration with the European Association for Cardio-Thoracic Surgery (EACTS): the Task Force for the diagnosis and management of atrial fibrillation of the European Society of Cardiology (ESC) Developed with the special contribution of the European Heart Rhythm Association (EHRA) of the ESC. Eur Heart J.

[2] Writing Committee Members, Joglar J.A., Chung M.K. (2024). 2023 ACC/AHA/ACCP/HRS guideline for the diagnosis and management of atrial fibrillation: a report of the American College of Cardiology/American Heart Association joint committee on clinical practice guidelines. J Am Coll Cardiol.

[3] Tzeis S., Gerstenfeld E.P., Kalman J. (2024). 2024 European Heart Rhythm Association/Heart Rhythm Society/Asia Pacific Heart Rhythm Society/Latin American Heart Rhythm Society expert consensus statement on catheter and surgical ablation of atrial fibrillation. Europace.

[4] Kuck K.H., Brugada J., Fürnkranz A. (2016). Cryoballoon or radiofrequency ablation for paroxysmal atrial fibrillation. N Engl J Med.

[5] Andrade J.G., Wells G.A., Deyell M.W. (2021). Cryoablation or drug therapy for initial treatment of atrial fibrillation. N Engl J Med.

[6] Wazni O.M., Dandamudi G., Sood N. (2021). Cryoballoon ablation as initial therapy for atrial fibrillation. N Engl J Med.

[7] Andrade J.G., Deyell M.W., Macle L. (2023). Progression of atrial fibrillation after cryoablation or drug therapy. N Engl J Med.

[8] Farkowski M.M., Karlinski M., Barra S. (2022). Effectiveness and safety of a single freeze strategy of cryoballoon ablation of atrial fibrillation: an EHRA systematic review and meta-analysis. Europace.

[9] Moser F., Rottner L., Moser J. (2022). The established and the challenger: A direct comparison of current cryoballoon technologies for pulmonary vein isolation. J Cardiovasc Electrophysiol.

[10] Tanese N., Almorad A., Pannone L. (2023). Outcomes after cryoballoon ablation of paroxysmal atrial fibrillation with the PolarX or the Arctic Front Advance Pro: a prospective multicentre experience. Europace.

[11] Honarbakhsh S., Martin C.A., Mesquita J. (2023). Atrial fibrillation cryoablation is an effective day case treatment: the UK PolarX vs. Arctic Front Advance experience. Europace.

[12] Tachibana S., Miyazaki S., Nitta J. (2024). Incidence of phrenic nerve injury during pulmonary vein isolation using different cryoballoons: data from a large prospective ablation registry. Europace.

[13] Knappe V., Lahrmann C., Funken M. (2025). Comparison of Arctic Front Advance Pro and POLARx cryoballoons for ablation therapy of atrial fibrillation: an intraprocedural analysis. Clin Res Cardiol.

[14] Reichlin T., Kueffer T., Knecht S. (2024). PolarX vs Arctic Front for cryoballoon ablation of paroxysmal AF: the randomized Compare CRYO study. JACC Clin Electrophysiol.

[15] Isonaga Y., Miyazaki S., Nitta J. (2024). Acute procedural efficacy and safety of a novel expandable diameter cryoballoon in atrial fibrillation ablation: early results from a multicenter ablation registry. J Cardiovasc Electrophysiol.

[16] Frommeyer G., Ellermann C., Wolfes J., Lange P.S., Güner F., Eckardt L. (2024). Feasibility and efficacy of a novel size adjustable cryoballoon for ablation of atrial fibrillation. J Interv Card Electrophysiol.

[17] Kawamura I., Miyazaki S., Inamura Y. (2024). A randomized controlled trial of the size-adjustable cryoballoon vs conventional cryoballoon for paroxysmal atrial fibrillation: the CONTRAST-CRYO II trial rationale and design. Heart Rhythm O2.

[18] Hayashi T., Hamada K., Iwasaki K., Takada J., Murakami M., Saito S. (2023). Difference in tissue temperature change between two cryoballoons. Open Heart.

[19] Smiseth O.A., Thompson C.R., Lohavanichbutr K. (1999). The pulmonary venous systolic flow pulse--its origin and relationship to left atrial pressure. J Am Coll Cardiol.

[20] Ghosh J., McGuire M.A. (2018). Atrial flow dynamics as a determinant of tissue temperature during balloon cryoablation. Europace.

[21] Ferreira A., Lobo L. (2009). Nitrous oxide: saturation properties and the phase diagram. J Chem Thermodyn.

[22] Mazur P. (1970). Cryobiology: the freezing of biological systems. Science.

[23] Parvez B., Goldberg S.M., Pathak V., Schubert C.M., Wood M.A. (2007). Time to electrode rewarming after cryoablation predicts lesion size. J Cardiovasc Electrophysiol.

[24] Gage A.A., Baust J.M., Baust J.G. (2009). Experimental cryosurgery investigations in vivo. Cryobiology.

[25] Erinjeri J.P., Clark T.W. (2010). Cryoablation: mechanism of action and devices. J Vasc Interv Radiol.

[26] Masuda M., Matsuda Y., Uematsu H. (2024). Impact of wall thickness on the tissue cooling effect of cryoballoon ablation. Europace.

[27] Sun W., Zheng L., Qiao Y. (2016). Catheter ablation as a treatment for vasovagal syncope: long-term outcome of endocardial autonomic modification of the left atrium. J Am Heart Assoc.

[28] Aksu T., De Potter T., John L. (2022). Procedural and short-term results of electroanatomic-mapping-guided ganglionated plexus ablation by first-time operators: a multicenter study. J Cardiovasc Electrophysiol.

[29] Penela D., Berruezo A., Roten L. (2024). Cardioneuroablation for vasovagal syncope: insights on patients’ selection, centre settings, procedural workflow and endpoints-results from an European Heart Rhythm Association survey. Europace.

[30] Armani Prata A., Katsuyama E., Scardini P. (2025). Cardioneuroablation in patients with vasovagal syncope: an updated systematic review and meta-analysis. Heart Rhythm.

[31] Ghosh J., McGuire M.A. (2013). Mitral regurgitation preventing pulmonary vein isolation by balloon cryoablation. Europace.

[32] Takami M., Misiri J., Lehmann H.I. (2015). Spatial and time-course thermodynamics during pulmonary vein isolation using the second-generation cryoballoon in a canine in vivo model. Circ Arrhythm Electrophysiol.

[33] Ghosh J., Sepahpour A., Chan K.H., Singarayar S., McGuire M.A. (2013). Immediate balloon deflation for prevention of persistent phrenic nerve palsy during pulmonary vein isolation by balloon cryoablation. Heart Rhythm.

[34] Mizutani Y., Yanagisawa S., Fujiwara G. (2023). Evaluation of the direction and extent of ice formation during cryoballoon ablation: an experimental study. J Interv Card Electrophysiol.

[35] Kawaji T., Bao B., Hojo S. (2024). Variation in the frozen lesion size according to the non-occluded application duration and technique for cryoballoon ablation. PLoS One.

