# Supplementary material for: Spatiotemporal tissue temperature during cryoablation using different balloons
Source: Heart Rhythm O2. 2025 Jun 16;6(9):1428–34. doi: 10.1016/j.hroo.2025.06.005 (PMC12635740; doi:10.1016/j.hroo.2025.06.005)
Supplement: Supplemental Video Legend [file mmc4.docx]

Video legends

**Video 1.**

Spatiotemporal tissue temperature evaluation during cryoablation using AFA-Pro.

**Video 2.**

Spatiotemporal tissue temperature evaluation during cryoablation using POLARx FIT 28 mm.

**Video 3.**

Spatiotemporal tissue temperature evaluation during cryoablation using POLARx FIT 31 mm.
